# Molecular Mediators of RNA Loading into Extracellular Vesicles

**DOI:** 10.3390/cells10123355

**Published:** 2021-11-30

**Authors:** Chiara Corrado, Maria Magdalena Barreca, Chiara Zichittella, Riccardo Alessandro, Alice Conigliaro

**Affiliations:** 1 Department of Biomedicine, Neuroscience and Advanced Diagnostics (Bi.N.D.), Section of Biology and Genetics, University of Palermo, 90133 Palermo, Italy; chiara.corrado@unipa.it (C.C.); mariamagdalena.barreca@unipa.it (M.M.B.); chiara.zichittella@unipa.it (C.Z.); riccardo.alessandro@unipa.it (R.A.); 2 Department of Biological, Chemical and Pharmaceutical Sciences and Technologies (STEBICEF), University of Palermo, 90128 Palermo, Italy; 3 Institute for Biomedical Research and Innovation (IRIB), National Research Council (CNR), 90146 Palermo, Italy

**Keywords:** exosomes, extracellular vesicles, non-coding RNA, miRNAs, lncRNAs

## Abstract

In the last decade, an increasing number of studies have demonstrated that non-coding RNA (ncRNAs) cooperate in the gene regulatory networks with other biomolecules, including coding RNAs, DNAs and proteins. Among them, microRNAs (miRNAs), long non-coding RNAs (lncRNAs) and circular RNAs (circRNAs) are involved in transcriptional and translation regulation at different levels. Intriguingly, ncRNAs can be packed in vesicles, released in the extracellular space, and finally internalized by receiving cells, thus affecting gene expression also at distance. This review focuses on the mechanisms through which the ncRNAs can be selectively packaged into extracellular vesicles (EVs).

## 1. Introduction

### 1.1. Extracellular Vesicles (EVs)

EVs are a family of membrane-coated vesicles with different proteomic and lipidomic profile, as well as different size.

In their study, Pan and Johnstone firstly described extracellular vesicles containing the transferrin receptor during reticulocytes maturation [1]. In the beginning, the scientific community thought that extracellular vesicles were containers of waste products of cellular metabolism that the cell wanted to remove. Nowadays the role of EVs is emerging as an important new area of biomedical research [2]. It is well known that EVs are actively secreted by cells and that their content can affect activities and functions of receiving cells, in physiological as well as in pathological conditions [3,4,5].

Due to their biogenesis, we can identify two different types of EVs: exosomes and microvesicles (MV) [6]. The last ones originate by direct budding of the plasma membrane with a rearrangement of the cytoskeleton [7]. On the contrary, the biogenesis and release of exosomes is a multi-step process. In brief, it begins with the inward budding of the plasma membrane to form the early endosome that, passing through the late endosome, maturates to multivesicular bodies (MVBs) [8]. These incorporate, through the endosomal sorting complexes required for transport (ESCRT), the intraluminal vesicles (ILs), which contain different cytosolic components, including proteins and nucleic acids. After the fusion of the MVBs with cell membrane, the ILs are released outside the cells as exosomes [9,10].

The different biogenesis of the EVs also affects the composition of the vesicles’ membrane. The invagination process of ILs determines a specific lipidic and protein composition of the exosome’s membranes, which gives them a distinctive molecular profile. Exosomes’ membranes contain ESCRT components and associated proteins, as TSG101 and Alix. Moreover, the participation of tetraspanins in exosomes formation determines an abundance of these proteins in the exo-membranes [11].

MV as shed directly from the plasma membrane, hold the annexin A1 [12] and are enriched in specific phospholipids such as phosphatidylserine (PS), which promotes uptake by recipient cells, or sphingolipids as well as ceramide and cholesterol, that allow MV formation [13].

Finally, EVs are characterized by their size; generally, microvesicles range from 200 to 2000 nm while exosomes from 30 to 150/200 nm. Recently the ISEV society established that it is better to prefer other methods of classification because many vesicles are quite similar in size range [14,15].

Many studies have focused on the biological effects of EVs in cell-cell communication both between neighboring cells and between cells in distant body districts. Biological fluids carry the EVs released in the extracellular space until they find a landing place where dump their content through the fusion of plasma membranes or endocytosis [16]. Recently, a great number of data support the transport of ncRNAs among cells, demonstrating that they can exert special functional roles [17]. This review aims to shed light and collect the latest published data about the mechanisms of RNA-loading in extracellular vesicles. To this end, we searched for papers indexed on the PubMed database by combining the following keywords: non-coding RNAs, EVs, and RNA-loading. The time limit was set from 2015 to date, however, we listed a few older documents since noteworthy as pioneering works. Among the emerged manuscripts, we summarized here those providing molecular and functional data on the RNA loading.

### 1.2. RNA Families in Extracellular Vesicles

Much of the recent interest in EVs was triggered by the discovery of their involvement in horizontal transfer of secreted extracellular RNA (exRNA). From the first evidence of functional mRNA in EVs, a huge number of studies revealed a significant assortment of ncRNAs in EVs. The deep sequencing RNA technique allows demonstrating a selective enrichment of small ncRNAs in human-derived extracellular vesicles, isolated from different cytotypes [18,19]. Among them, small RNA families are the most abundant, these include small nuclear RNAs, small nucleolar RNAs, ribosomal RNAs, transfer RNAs, miRNAs. However, also larger RNAs groups, including mitochondrial RNAs, Y RNA, vault RNA, piwi RNA and long non-coding RNA have been found in EVs, for a detailed review of the different RNAs families in EV see [20]. A recent work of Mosbach et al. demonstrated that RNA sorting depends on its size but also on its origin, the authors in fact revealed that RNA polymerase III transcripts are preferentially associated with EVs [21].

**Figure 1:**
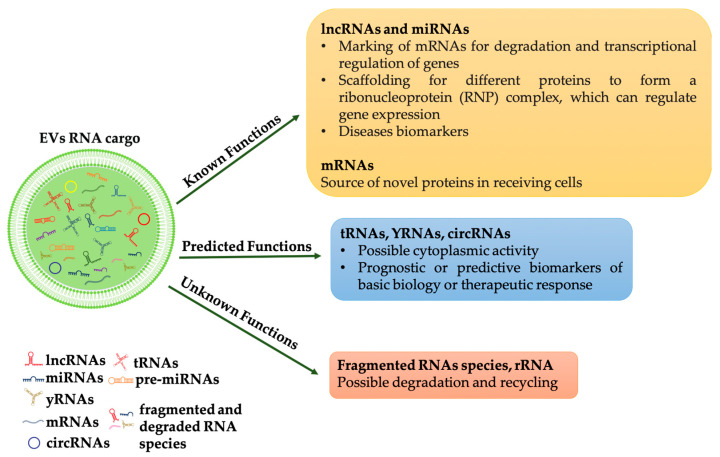
RNAs species into EVs and their functions. RNAs in extracellular vesicles can be classified into three types: (1) RNAs that have known function when internalized into target cells, such as mRNAs, miRNAs and lncRNAs; (2) RNAs that are predicted to be functional (for example, tRNAs, YRNAs, circRNAs); (3) RNAs with unknown functions (for example, fragments RNAs and rRNAs), some of which may be functional, but others may be non-functional degradation products.

As pointed out by [20] although several types of RNAs have been identified in EVs, only some of these have been demonstrated functioning in the recipient cell, i.e., miRNAs and lncRNAs. For other RNA molecules, such as tRNAs, can be assumed a possible activity in the cytoplasm of the recipient cells while the role of other RNA molecules such as mRNA fragments or ribosomal RNAs remains unclear (Figure 1).

Still controversial is the presence of miRNA precursors: their identification, together with Dicer and Argonaute 2, let scientists to suppose that complete silencing machinery could be transferred by EVs [22,23]. However, the hypothesis was not largely confirmed by other investigators.

Other conflicting observations in studying exRNAs are due to the different isolation strategies. For example, ultracentrifugation, the gold standard method for EVs purification, biases RNAs data. It in fact does not allow to separate vesicles from free ribonucleoproteins or lipoproteins both associated with RNAs [24].

To overcome this limitation, Lässer group developed a method combining size exclusion chromatography with a density cushion; this allows to isolate and characterize EVs from blood with minimal contamination by plasma proteins and lipoprotein particles [25].

Meanwhile, Jeppesen et al. used high-resolution density gradient fractionation to separate EVs from non-vesicular material, then, with a direct immunoaffinity capture (DIC) targeting classical exosomal tetraspanins, exosomes were specifically isolated from other types of EVs [12]. Nucleic acid analysis revealed that exRNAs are differentially expressed between EVs and non-vesicle compartments. Interestingly the authors demonstrated that miRNAs were mainly associated with extracellular non-vesicular fractions while the EVs were enriched in transfer RNA (tRNA) fragments. In addition, YRNA and vault RNA were particularly enriched in non-vesicular fractions [12].

New isolation techniques have allowed more accurate identification of the RNA families transported through the EVs, further confirming the role of vesicles in the horizontal transfer of this nucleic acid.

## 2. The Effects of the Horizontal Transfer of EVs Derived RNAs

Many studies demonstrated the functional role of the RNA-cargo that, selectively packaged inside EVs, are transferred to target cells. Firstly, the transfer of exosomal mRNAs and miRNAs was reported by Valadi et al. in 2007, which revealed a “novel mechanism of genetic exchange between cells” [26]. Since that study, numerous papers have demonstrated that the horizontal transfer of exosomes cargo can modulate target cells behaviors.

Studies on tumor cells revealed as exosome-mediated RNA transfer may control tumor growth, affect its microenvironment, and promote metastases.

It is well known that a central event in tumor progression is the induction of angiogenesis. Focusing on this pathway it has been demonstrated that glioblastoma, chronic myelogenous leukemia, and breast cancer derived EVs can reprogram endothelial cells through horizontal transfer of their miRNAs cargo [27,28].

The involvement of cancer derived EVs in facilitating brain infiltration is worth mentioning. Lu and collaborators have recently demonstrated that exosomes derived by highly brain metastatic breast cancer cells are able to destroy the blood–brain barrier through its lncRNA *GS1-600G8.5* [29,30].

Exosomal miRNAs have a role also in tumor drug resistance. Mao et al. support this hypothesis demonstrating that Adriamycin-resistant breast cancer cells deliver specific miRNAs through exosomes thus promoting drug resistance in neighboring cells [31]. Quin and collaborators demonstrated that exosomes derived from cisplatin-resistant lung cancer cell line A549 induce drug resistance in receiving cells. MiRNA profile identified the *miR100-5p* as the mediator of this process [32].

In addition, exosome-transported lncRNAs may participate in drug resistance induction. A recent paper by Wang et al. proved that the exosome-mediated transfer of lncRNA *H19* induces doxorubicin resistance in breast cancer [33]. The authors showed that drug-resistant cells release exosomes enriched in *lncH19*; these exosomes increase the chemoresistance of doxorubicin once internalized by sensitive cells. Moreover, downregulation of *H19* in sensitive cells ablated this effect thus confirming the direct role of the long non-coding RNA [33]. EVs released into the tumor microenvironment strongly affect metastatic niche. For example, the prometastatic miRNA *miR-9* and *miR-155*, carried respectively by breast cancer derived exosomes and pancreatic cancer derived microvesicles, are able to reprogram fibroblast to cancer associated fibroblast (CAF) phenotype thus promoting tumor progression [34,35]. While the EV-mediated delivery of the *miR-105* and -*miR-122* reprogram CAFs metabolism to sustain tumor growth [36,37]. Moreover, cancer derived EVs can reprogram immune cells thus preventing immunosurveillance and promoting immunotolerance in cancer microenvironment, as revised by Graner [38]. In addition, EVs derived RNAs profiling can be useful as a prognostic indicator to therapeutic response. For example, *miR196a-5p* and *miR-501-3p* were significantly downregulated in exosomes isolated from the urine of prostate cancer patients [39], while *let7-b* and *miR-18a*, isolated from plasma of multiple myeloma patients, were associated with overall survival [40]. As expected, the effects of the EV-transported RNA are not limited to the tumor context.

Barile and colleagues revealed the cardioprotective role of the different miRNAs transported by EVs derived from cardiac progenitor cells (CPC). They demonstrated that *miR-210* inhibits cardiomyocyte apoptosis by targeting Ephrin A3 (cell surface GPI-bound ligand for Eph receptors) and PTP1b (protein-tyrosine phosphatase 1b). Moreover, EVs derived *miR132* stimulates angiogenesis acting on RasGAP-p120, Ras GTPase activating protein p120 [41]. Furthermore, CPC derived exosomal *miR21* exert similar effects preventing cell apoptosis by targeting PDCD4 (Programmed Cell Death 4) [42]. Similarly, Gray and collaborators, through microarray analysis of exosomes derived from hypoxic CPC, identified 11 miRNAs that improve cardiac function stimulating tube formation of endothelial cells and reducing fibrosis [43].

Regarding differentiation, it has been demonstrated that exosomes released from Neural stem/progenitor cells (NPCs) have an important role in neurogenesis; Ma and collaborators demonstrated that mouse cortical NPCs, isolated from fetal brain, promote neuronal differentiation through exosomal *miR-21a* [44].

Adipose tissue is an excellent resource for circulating exosomal miRNAs that have a role in regulating liver gene expression, as well as affecting obesity or diabetes. Adipose-derived exosomal *miR-99b* has been demonstrated to control in vivo fibroblast growth factor 21 (FGF21) production [45]. While exosomes secreted by adipose tissue macrophages transfer miRNAs modulating, in vivo and in vitro, insulin sensitivity and glucose homeostasis [46].

**Figure 2:**
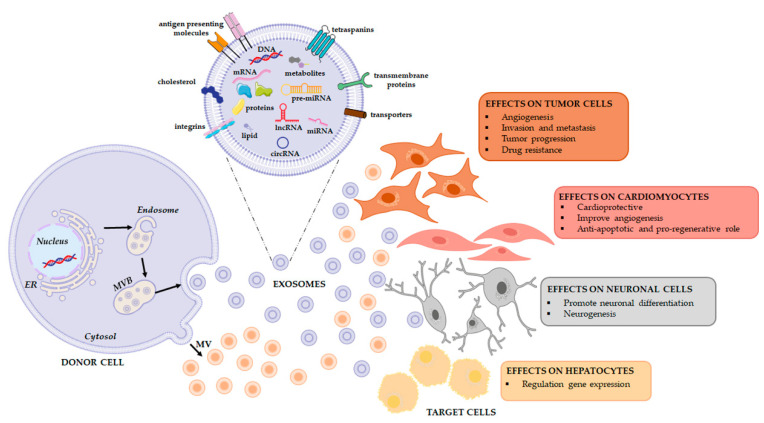
Extracellular vesicles release and their functional effects on target cells. EVs are a heterogeneous population, both in form and content since their cargo is strictly dependent on the pathophysiological conditions of the cell at the exact moment in which it produces the vesicle. When studying the complexity of the EV-mediated cell-cell communication, it is necessary to evaluate that the same vesicle, i.e., the same message, can be interpreted differently depending on the cytotype that receives it. This will depend in good part on the gene expression profile of the recipient cell.

All these experiments suggest that exosomes may be a vehicle of therapeutic non-coding RNAs in physiological and pathological conditions as well as in the field of regenerative therapy (Figure 2) (Table 1). To this end, a comprehensive analysis of the RNA loading mechanisms is appropriate.

## 3. Loading of EVs and Cargo Sorting

### 3.1. RNA Binding Protein-Mediated Loading

Recent evidence highlighted the main role of RNA-binding proteins in RNA sorting and loading in EVs. Santangelo et al. have identified the RNA binding protein SYNCRIP (synaptotagmin-binding cytoplasmic RNA-interacting protein; also known as hnRNPQ or NSAP1) as a component of the hepatocyte exosomal miRNA sorting machinery. They showed that SYNCRIP knockdown impairs the internalization of miRNAs in exosomes [47]. Subsequently, Hobor et al., identified that SYNCRIP contains a sequence called NURR (N-terminal unit for RNA recognition) that recognizes and bind the motif GGCU/A in miRNAs. This interaction guides miRNA loading into exosomes [48].

Recently, Temoche-Diaz and collaborators showed that the metastatic breast cancer cell line MDA-MB-231 releases two sub-populations of EVs, called vesicular low density (vLD) and vesicular high density (vHD) EVs. Mass spectrometry analysis revealed that CD63, known as an exosomal marker, was enriched in the vHD subpopulation, as well as other endosome-associated proteins. MiRNA analysis identified five miRNAs that were enriched in vHD vesicles and not in vLD ones, thus suggesting that there is a specific mechanism of miRNA sorting for the vHD subpopulation [49]. Interestingly, *miR-122*, a well-known prognostic biomarker for metastasis in breast cancer patients [50,51] was identified. The authors demonstrated that the RNA binding protein Lupus La drives the selective *miR-122* vHD enrichment in breast cancer cell lines [49].

RNA binding proteins not only promote RNA loading but also contribute to RNA function into target cells. Chen et al. studying bladder cancer metastatization identified an exosomal lncRNA, termed lymph node metastasis-associated transcript 2 (*LNMAT2*) that promotes lymphangiogenesis. Interestingly, they found that *LNMAT2* was loaded into exosomes by the heterogeneous nuclear ribonucleoprotein A2/B1 (HNRNPA2B1). The complex *LNMAT2*/hnRNPA2B1, once in receiving cells, can migrate into the nucleus and interact with PROX1 promoter. Here HNRNPA2B1 mediates H3 lysine 4 trimethylation (H3K4me3) and subsequent activation of PROX1 which, in turn, regulates endothelial cell differentiation and metastatic dissemination [52].

Often post-translational modifications of RNA binding proteins, such as SUMOylation, phosphorylation or glycosylation, contribute to regulating exosome loading and release [53,54].

Regarding non-coding RNAs loading, the sumoylation of heterogeneous nuclear ribonucleoproteins (HNRNPs) is one of the proposed mechanisms. HNRNPA1, after sumoylation, recognizes the long non-coding RNA *ELNAT1* thus mediating its packaging into EVs [55]. While HNRNPA2B1, after sumoylation, binds miRNAs recognizing the EXOmotif GGAG (conserved motifs that allow the specific miRNAs sorting into exosomes) and this interaction is responsible for miRNAs loading into exosomes [56].

The ribonucleoprotein HNRNPA2B1, again, through GlcNAcylation, can regulate the internalization of specific miRNAs into EVs. Recently, Lee and collaborators demonstrated that Caveolin1 (CAV1), after its tyrosine phosphorylation (Y14), interacts with HNRNPA2B1, promoting HNRNPA2B1 O-GlcNAcylation on two serine domains. This modified complex directly controls the packaging of specific miRNAs into EVs [57]. Another study by McKenzie and colleagues highlighted that Ago2 phosphorylation could control loading of miRNAs in exosomes [58].

In a recent work, Robinson and coworkers have identified HNRNPK as a CAV1-regulated microRNA binding protein. They observed that CAV1 drives HNRPNK localization to MVB, which brings the miRNA containing the AsUGnA motif. Moreover, they found that membrane-rafts take part in this transport [59]. Export of miRNAs into exosomes and their subsequent release, in fact, can occur also through a lipid rafts dependent mechanism, as discussed later.

Again, HNRNPK participates in EVs loading and secretion of non-coding RNAs, through an alternative pathway mediated by LC3-conjugation machinery and called LDELS, LC3 dependent EV loading and secretion [60].

Leidal et al. demonstrated that lipidated LC3-II is involved in the loading of specific proteins into intraluminal vesicles (ILVs) for their subsequent release as EVs. Interestingly the process is distinct from classical macroautophagy/autophagy because it requires components of the LC3 conjugation machinery, but not other ATGs involved in autophagosome formation. By proteomic analyses of LC3-conjugated protein obtained from secretome, the authors demonstrated that 33% of identified proteins have been previously associated with EVs. Moreover, after sucrose density gradient purification, they found that endogenous LC3-II co-fractionated with well-defined EV markers. This result further confirms the presence of LC3-II residues inside the lumen of EVs. Through different knock out models, the authors identified several RNA binding proteins that require LC3 to be internalized in EVs, among these the HNRNPK and scaffold-attachment Factor B (SAFB). Interestingly the deficiency in LC3-conjugation machinery affects the amounts of RNA loaded in EV. In particular, snoRNAs and miRNAs were reduced with an increase in tRNAs while no effects were found in large RNA levels [60].

New evidence from Arabidopsis proteomic analysis revealed that also plant- derived EVs contain several RNA-binding proteins, as Argonaute 1 (AGO1) and RNA helicases (RHs). These proteins selectively associate with EV-enriched small RNAs, thus suggesting their involvement in specific loading of sRNAs into EVs also in plants [61].

**Figure 3:**
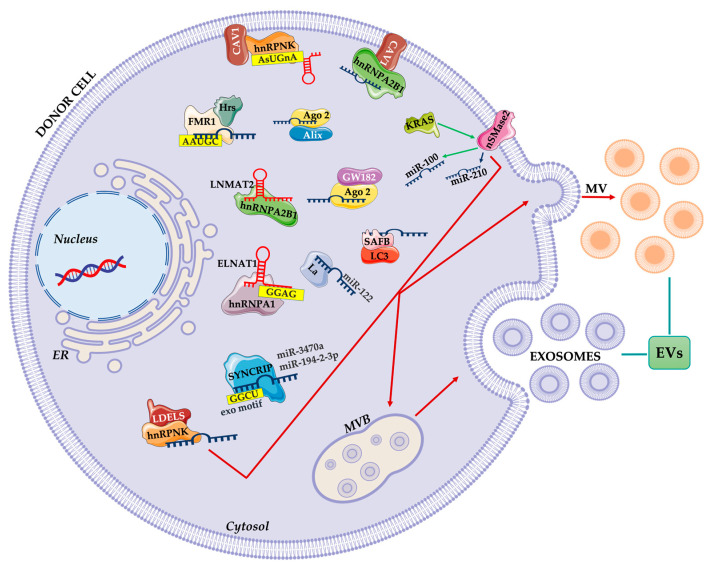
Summary of proteins involved in the ncRNA packaging into EVs. RNA binding proteins alone or in cooperation with other proteins bind specific ncRNAs and selectively transport them into EVs. Membrane proteins are also involved in the ncRNA loading EVs mechanism.

In conclusion, several RNA binding proteins, alone or in combination with other molecular interactors may control RNA sorting inside EV (Figure 3).

### 3.2. Other Carriers for RNA Loading

It is well known that the ESCRT pathway is responsible for protein sorting into EVs [62,63].

Alix is an adaptor protein involved in EVs biogenesis and cargo sorting through an ESCRT dependent pathway [64]. However, new evidence indicated that Alix is involved in miRNAs loading into EVs. Co-immunoprecipitation experiments revealed a direct interaction between Alix and the RNA binding protein Ago2, commonly involved in miRNA transport and processing. This complex drives Alix with Ago2-associated miRNAs into EVs [65,66]. A further connection between ESCRT complex and selective RNA loading was confirmed by Wozniak et al. [67]. In particular, the authors demonstrated that the RBP fragile X mental retardation 1 (FMR1) interacts with the hepatocyte growth factor-regulated tyrosine kinase substrate (Hrs), a component of the ESCRT complex. The RNA binding protein FMR1 acts as a chaperone that recognizes a specific sequence in miRNA (AAUGC) while Hrs allows complex internalization. Interestingly, inflammosome activation mediates this interaction, through the cleavage of the trafficking adaptor protein RILP (Rab-interacting lysosomal protein) that works as ride [67].

Cargo sorting could also be driven via ESCRT-independent pathways, e.g., through the neutral sphingomyelinase, phospholipase or other lipids and associated protein such as tetraspanin [9,10,60,68].

Janas et al. have proposed that the RNA loading into exosomes could be mediated by the direct interaction between RNAs and the lipid raft of MVB membrane [69]. Moreover, in their recent studies, through bioinformatic analysis, they identified four raft RNA motifs that are frequent in the exosomal pro-tumoral miRNAs transferred from cancer cells to immune cells [70].

Other recent evidence suggests that neutral sphingomyelinase 2 (nSMase 2), via ceramide production, regulates EVs cargo loading. Kosaka and coworkers demonstrated that nSMase2 promotes the *miR-210* packaging in EVs that finally affect the initiation of metastatic process through the induction of angiogenesis [71]. Cha et al. compared small RNAs from isogenic cell lines, that differ only in KRAS status, to demonstrate a KRAS dependent sorting of miRNAs into EVs. In mutant KRAS-derived EVs authors showed enrichment of tumor-suppressive *miR-100* while the oncomiR *miR-10b* was preferentially secreted in EVs derived from wild type KRAS cell line; these data demonstrate a key role for KRAS in orchestrating RNA trafficking. Interestingly nSMase2 is the mediator of this mechanism since its inhibition caused the *miR-100* confinement in KRAS mutated cells [72].

Sphingomyelinase takes part also in the transport of viral RNA mediated by EVs. Zhou et al. demonstrated that ZIKA viruses, which infection induces severe neurological manifestations, use exosomes as mediators of viral transmission between neurons. The authors demonstrated that cortical neuronal cell-derived exosomes contain ZIKA virus RNAs and proteins and that the treatment with GW4869, nSMase-2/SMPD3 specific inhibitor, significantly reduced the loading of viral molecules. Interestingly, viral RNA positively participates in transmission promoting in neuronal cells the expression and activity of the nSMase-2 [73].

Finally, it has to consider the possibility that the RNAs are not passively loaded into the exosomes but may themselves participate in vesicle formation. This is the case of lncRNA plasmacytoma variant translocation 1 (*PVT1*) that, highly expressed in several cancers including pancreatic cancer, promotes exosome secretion [74]. Sun et al. demonstrated that *PVT1* promotes the docking of MVBs by altering RAB7 expression and localization. Moreover, *PVT1* promotes the palmitoylation of the v-SNARE homolog YKT6 and its colocalization with vesicle-associated membrane protein 3 (VAMP3) thus determining the fusion of MVBs with the plasma membrane. The involvement of lncRNA in exosomes formation or release let also suppose a direct role of these molecules in transporting other smaller RNAs.

In conclusion, cargo sorting could be mediated by carriers commonly involved in EV biogenesis or in miRNA transport and processing. Cargo sorting could also be driven via ESCRT dependent or via ESCRT-independent pathways, e.g., through the neutral sphingomyelinase, phospholipase or other lipids and associated proteins (Figure 2).

### 3.3. Engeneering Vesicles for RNA Loading

Several characteristics of EVs make them an interesting candidate for RNA delivery. EVs in fact can cross biological barriers, avoid toxicity and immunogenicity, and have an endogenous targeting ability. However, not all EVs show the same properties.

A recent paper by Murphy et al. formally demonstrated that EVs possess higher RNA delivery efficiency than synthetic RNA delivery systems, used for clinical delivery of siRNAs [75]. To evaluate the efficiency of transported RNAs, the authors developed the CRISPR Operated Stoplight System for Functional Intercellular RNA Exchange (CROSS-FIRE) reporter system that can be activated only by the functional transfer of a specific single-guide RNA (sgRNA). Interestingly, comparing the sgRNA-delivery among EVs and synthetic systems the authors confirmed the highest efficiency of EVs. Moreover, comparative analyses among EVs from different cell lines highlighted the divergence in the efficiency of encapsulating RNA by up to 30 times [75].

Several groups are developing engineering strategies to facilitate loading to develop systemic delivery of siRNA or miRNA by EVs. The ultra-thermostable pRNA-3WJ core has been used in many applications including gene therapy, target specific delivery, controlled drug release, and image-guided diagnostics [76]. Recently Pi et al. demonstrated that the arrow-shaped pRNA-3WJ offers the opportunity to control either partial loading of RNA into EVs or decoration of ligands on the surface of EVs. With cholesterol placed on the arrow-tail of the 3WJ, in fact, the RNA is mainly located on the surface of the EVs, while, placing the cholesterol at the arrowhead resulted in partial loading of RNA nanoparticles into the extracellular vesicles [77].

Specific loading of miRNA or siRNA in EVs has been performed through overexpression of the interested RNA in EV producing cells; however, recently the manuscript of Sork et al. demonstrated this strategy as a “wasteful way of loading miRNA to EVs”. The authors transiently overexpressed two different pri-miRNAs in HEK293-T cells and quantified the respective mature miRNA levels in the EV and non-EV portion separated by size-exclusion chromatography. The analysis revealed that most overexpressed mature transcripts were secreted in the non-EV fraction, whereas the EV fraction contained <2% of the respective miRNAs [78].

The new generation of loading techniques uses RNA binding proteins to guide the internalization of specific RNA sequences. Kojima et al. [79] among EXOtic devices able to boost exosome production and application, developed a specific RNA packaging system using the archaeal ribosomal protein L7Ae which binds to the C/D box RNA structure. They conjugated L7Ae to the C-terminus of CD63, thus allowing its localization on the exosomal membrane, and inserted a C/D box into the 3′-untranslated region (3′-UTR) of the reporter gene [79].

Wang et al. took advantage of the use of the transactivator of transcription (Tat) protein, which binds specifically to the stem-loop-containing trans-activating response (TAR) element RNA. They added TAR directly to the 5′ end of a cargo mRNA and fused Tat peptide directly to the C-terminus of ARRDC1, the Arrestin domain containing protein 1 [80].

Despite numerous attempts to engineer vesicles with specific RNAs, the major limitation is still the partial knowledge of the sorting and loading mechanisms in nature.

## 4. Conclusions

Recent investigations have shown the central role of ncRNAs in gene regulation at different levels, including the ‘remote’ regulation mediated by EVs.

Here we summarized the newest studies describing the RNA binding proteins and other interactors controlling the RNA sorting inside EVs. However, many aspects of RNA trafficking need to be further investigated. Moreover, it remains unclear why cells selectively load specific RNAs into subclasses of extracellular vesicles unless we still want to convince ourselves that it is only a strategy for discarding excess materials.

Detailed knowledge of the loading mechanisms may point towards the wider use of EVs as diagnostic and prognostic tools. Moreover, it can provide essential indications for enhancing the use of EVs as drug delivery systems.

## Figures and Tables

**Table 1:** Functional effects of ncRNAs shuttled by extracellular vesicles.

EVs Derived ncRNAs	Donor Cells/Tissues or Biofluids	Target Cell/Tissues or In Vivo Model	Effect	References
miR-20a, miR-23a, miR-24, miR-149 and miR-222	Drug-resistant breast cancer cells variant (MCF-7/Adr)	Drug-sensitive human breast cancer (MCF-7/S)	Promote drug resistance	[31]
miR-100-5p	Cisplatin resistant lung cancer cells (A549/DDP)	A549 lung cancer cells and tumor tissues of BABL/c athymic nude mice	Induces drug resistance	[32]
lncRNA H19	MCF-7/DOX and MDA-MB-231/DOX (DOX-resistant breast cancer cells)	MCF-7 and MDA-MB-231 (Sensitive breast cancer cells)	Induces drug resistance	[33]
miR-9	Breast cancer cells (MDA-MB-231 and MDA-MB-468)	Human breast Fibroblasts (NFs)	Promotes tumor progression by inducing properties similar to the CAF phenotype	[34]
miR-155	Pancreatic cancer cell lines (BxPC-3 and SW1990)	Primary pancreatic fibroblasts from wild type C57 mice	Reprograms the phenotype of normal fibroblasts in CAF	[35]
miR-105	Breast cancer cells (MDA-MB-231)	Patient-derived primary fibroblasts (CAF265922)	Reprograms the metabolism of CAFs to support tumor growth	[36]
miR-122	Breast cancer cells (MDA-MB-231)	Mouse primary lung fibroblasts	Reprograms glucose metabolism in the premetastatic niche to promote metastasis. Predictive marker and possible therapeutic target for metastatic BC	[37]
miR196a-5p and miR-501-3p	Urinary exosomes from prostate cancer patients (Pca)		Non-invasive prognostic biomarkers for prostate cancer	[39]
let7-b and miR-18a	Exosomes from plasma of multiple myeloma patients (MM)		Predictors of progression-free survival (PFS) and overall survival (OS) in patients with MM	[40]
miR-210	CPCs	Mouse cardiomyocytic cells (HL-1)	Cardioprotective role, inhibits cardiomyocyte apoptosis	[41]
miR-132	CPCs	HUVECs	Anti-apoptotic and pro-angiogenic role, enhancing tube formation ability of endothelial cells	[41]
miR-21	CPCs	H9C2 (human cardiomyocytic cells)	Anti-apoptotic role, preventing apoptosis of cardiomyocytes	[42]
miR-15b, miR-17, miR-20a, miR-103, miR-199a, miR-210 and miR-292	CPCs in hypoxic conditions	Rat primary cardiac microvascular endothelial cells (CECs) and rat cardiac fibroblasts	Pro-regenerative role;—promote cardiac function by stimulating tube formation of the endothelial cell and reducing fibrosis	[43]
miR-21a	NPCs	NPCs	Promotes neurogenesis and neuronal differentiation	[44]
miR-99b	Adipose tissue	Distant tissues	Increases in vivo hepatic FGF21 expression Increase glucose tolerance	[45]
miR-155	ATMs (adipose tissue macrophages) in leads mice	Obese insulin resistant mice	Modulation of insulin sensitivity and glucose homeostasis	[46]
